# Vascular Function Recovery Following Saturation Diving

**DOI:** 10.3390/medicina58101476

**Published:** 2022-10-17

**Authors:** Jean-Pierre Imbert, Salih-Murat Egi, Costantino Balestra

**Affiliations:** 1 Environmental, Occupational, Aging (Integrative) Physiology Laboratory, Haute Ecole Bruxelles-Brabant (HE2B), 1090 Brussels, Belgium; 2 Divetech, 1543 Chemin des Vignasses, 06410 Biot, France; 3 Computer Engineering Department, Galatasaray University, Istanbul 34349, Turkey; 4 Anatomical Research and Clinical Studies (ARCS), Vrije Universiteit Brussels (VUB), 1090 Brussels, Belgium; 5 DAN Europe Research Division, 64026 Roseto, Italy; 6 Physical Activity Teaching Unit, Motor Sciences Department, Université Libre de Bruxelles (ULB), 1050 Brussels, Belgium

**Keywords:** flow-mediated dilation, FMD, decompression, arterial stiffness, endothelial dysfunction, underwater, hyperbaric, commercial diver, off-shore energy operation, human

## Abstract

*Background and Objectives*: Saturation diving is a technique used in commercial diving. Decompression sickness (DCS) was the main concern of saturation safety, but procedures have evolved over the last 50 years and DCS has become a rare event. New needs have evolved to evaluate the diving and decompression stress to improve the flexibility of the operations (minimum interval between dives, optimal oxygen levels, etc.). We monitored this stress in saturation divers during actual operations. *Materials and Methods*: The monitoring included the detection of vascular gas emboli (VGE) and the changes in the vascular function measured by flow mediated dilatation (FMD) after final decompression to surface. Monitoring was performed onboard a diving support vessel operating in the North Sea at typical storage depths of 120 and 136 msw. A total of 49 divers signed an informed consent form and participated to the study. Data were collected on divers at surface, before the saturation and during the 9 h following the end of the final decompression. *Results*: VGE were detected in three divers at very low levels (insignificant), confirming the improvements achieved on saturation decompression procedures. As expected, the FMD showed an impairment of vascular function immediately at the end of the saturation in all divers but the divers fully recovered from these vascular changes in the next 9 following hours, regardless of the initial decompression starting depth. *Conclusion*: These changes suggest an oxidative/inflammatory dimension to the diving/decompression stress during saturation that will require further monitoring investigations even if the vascular impairement is found to recover fast.

## 1. Introduction

Saturation diving is a standard technique of divers’ intervention in the North Sea because of its depth (average 100 to 150 msw). Saturation is conducted from large diving support vessel that employs around 80 divers in multiple rotations during a working season. The contractors have developed saturation procedures empirically over the last 40 years and reached a mature level of technology and safety. On the other hand, the need for evaluating the diving and decompression stress to improve flexibility of the operations (minimum interval between dives, optimal oxygen levels, etc.) arose.

An increasing number of research reports have been published to document procedures, diver’s subjective evaluations [1], hematological changes [2], high pressure nervous syndrome [3], divers hydration status [4] or oxidative stress [5]. Saturation permits divers to live under pressure in chambers onboard of a vessel and to be deployed directly to the seabed by a diving bell. Historically, commercial saturation diving was developed during the 1970s for the North Sea oil platform installations. At the time, the concern was decompression sickness (DCS) that was associated with bubbles in the divers’ blood. During these “early days” when diving in such a harsh environment was not as safe, and still difficult today, alarming reports were available:

*“Records show that since 1966 seventy-seven diving personnel tragically have lost their lives in the quest for, depending on your perspective “Black Gold” or “Devil’s excrement” in the North Sea Basin. By nationality: 53 British/Commonwealth subjects, 9 American, 7 Norwegian, 4 Dutch, 3 French, and 1 Italian. Prior to 1971/1974, applicable laws & regulations (if any) required no accurate fatal accident statistics. One can conclude that the actual combined number of deaths is higher. However, it is known that several divers received severe injuries from which they never recovered.”*

(https://the-norwegian.com/north-sea-diving-fatalities (accessed on 20 September 2022))

Fifty years later, saturation procedures have improved a lot and decompression sickness has become a rare event. Official safety records published in Norway on the Website of the PSA (Petroleum safety Authority) indicate an incidence of less than one case per 2000 exposure over the last 10 years (https://www.ptil.no/en/technical-competence/explore-technical-subjects (accessed on 20 September 2022)). As a result, the diving companies have the duty to evaluate the performances of their procedures such as the minimal permitted interval between two saturations. This minimum interval has been arbitrarily defined for a long time by industry guidelines or diving regulations but the divers’ recovery between saturations has never been studied scientifically. This recovery period remains important for companies to optimize their crew changes and divers to manage their professional career.

Saturation diving is obviously associated with multiple stressors that may be organized along three dimensions for simplicity. The first dimension is characterized by the diving work and includes stresses such as the physical, mental, or thermal.

The second dimension is associated with the vascular gas emboli (VGE) produced during decompression. Although there is no clear relation between the number of VGE measured and the risk of DCS, it is recognized that the smaller the number of VGE detected, the safer is the decompression [6]. The number of circulating VGE was therefore taken as the principal measurement of the decompression stress.

The third dimension covers several biological processes recently identified in the literature [7]. New insight demonstrate that bubbles tear the vessel inner layer away and create microparticles of endothelial debris when detaching from the endothelium during decompression [8,9,10]. Bubbles and oxygen partial pressure increase trigger defense mechanisms like platelets and neutrophil activation that will also elicit some microparticles [11,12]. In this study, the vascular function assessed by means of Flow Mediated Dilation (FMD) was considered as the third dimension representing the oxidative and or inflammatory stress [10,13,14].

The objective of the study was to define a monitoring package and use it on board a vessel to monitor saturation divers at surface, before the saturation and after exiting saturation (after decompression), in order to evaluate their recovery during the 9 h following the end of their final decompression.

## 2. Methods

### 2.1. Worksites

**Figure 1:**
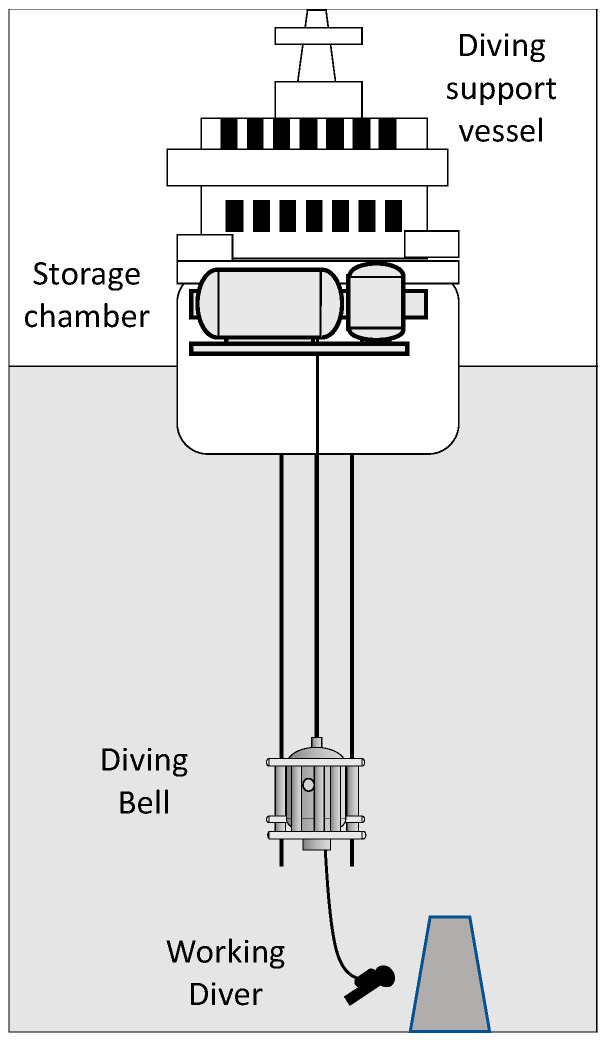
A typical saturation worksite. The divers are deployed from the diving support vessel inside a diving bell. Once on site, the bell’s door opens, and the divers lock out in the water using an umbilical attached to the bell to breathe and being supplied with hot water in their suit for thermal comfort. The working depth corresponds to the maximum depth reached by the divers. The working depth defines the chamber storage depth from excursion tables prepared in the company diving manual. The bell depth is usually set at 5 msw deeper than the storage depth to clear from subsea structures when opened. The “storage” and the “bell” are almost at the same pressure allowing for getting back to storage after work without decompression needed. The excursion of the diver out of the diving bell is limited to some meters not to add additional decompression time. The breathing gas is Heliox (Helium-Oxygen) to limit the density of the breathed gas (significant at such pressures) to reduce the work of breathing as well as Oxygen toxicity and Nitrogen narcosis.

A leading diving company provided access to one of their diving support vessels (DSV) operating in the North Sea for this study. Two monitoring sessions were conducted onboard the DSV Deep Arctic (The vessel DEEP ARCTIC is an Offshore Support Vessel built in 2009 with particulars of Gross Tonnage 18,640 t; Summer Deadweight 13,000 t; Length Overall 157 m; Beam 31 m.) in April and October 2016, during two different projects, one in the Norwegian sector at 121 m of sea water (msw) and the other in the UK sector at 155 msw working depth. The two projects, performed on the same vessel, corresponded to a well intervention on the seabed; the divers used the same breathing gasses, the same diving equipment and performed the same tasks (see Figure 1).

### 2.2. Saturation Procedures

The two projects were conducted with saturations according to the Company procedures defined in their diving manuals. However, specific requirements are defined in the Norwegian diving regulations that introduced slight variations.

The chambers were initially compressed to 10 msw in 10 min for a 20 min leaks check. Compression then proceeded to the “storage” depth at 1 msw/min.

The chamber PO_2_ at storage depth was controlled at 40 hPa. The storage depth was selected from the working depth using the standard excursion tables (110 msw storage depth for 121 msw working depth in the Norwegian sector, 136 msw storage depth for 155 msw working depth in the UK sector). During the bottom phase, divers performed one bell dive of 8 h per day but may sometime skip a dive due to weather conditions or vessel transit. During the dives, the divers’ breathing mixture was Heliox with a PO_2_ ranging from 60 to 80 hPa.

The final decompression can only start after an 8 h period following the last excursion dive.

The decompression is performed in two phases. It starts with constant chamber PO_2_ (50 hPa in the UK, 48 hPa in Norway) until 15 msw and finishes with a chamber oxygen percentage maintained between 23.1 and 23% to limit the fire hazard and optimize inert gas exhalation.

Despite the difference between sectors, the total decompression durations were very similar (5 days 5 h in the UK sector and 5 days 11 h in the Norwegian sector, a difference of less than 3%).

The divers were organized in three men teams (two divers and the bellman). Teams worked in shifts (12:00 p.m. to midnight and midnight to 12:00 p.m.). Each team was involved in one bell excursion dive per day during their shift. The divers’ in-water time was limited to 6 h with a mandatory break at mid-excursion.

### 2.3. Participant Eligibility and Enrollment

The study group consisted of volunteer, male, certified commercial saturation divers. These divers were declared fit for the saturation by the vessel hyperbaric nurse after a mandatory pre-dive medical examination.

All experimental procedures were conducted in accordance with the Declaration of Helsinki [8] and were approved by the Academic Ethical Committee of Brussels (B200-2009-039). The methods and potential risks were explained in detail to the participants. Each subject gave written informed consent before participation.

A total of 49 divers accepted to participate to the study.

The group anthropometric parameters were obtained after a confidential interview in the vessel hospital. (See Table 1).

As expected from saturation divers, all were very experienced divers with a long diving career. (See Table 2).

Part of the group freely took of antioxidant supplements (commercially available products containing as vitamin C, D, or E) before and during the saturation. (See Table 3).

Saturation divers generally spend a lot of time maintaining a high level of physical fitness and are involved in all sorts of sports. Every diver in the group except one had a daily or at least weekly physical activity when at home. (See Table 4).

**Figure 2:**
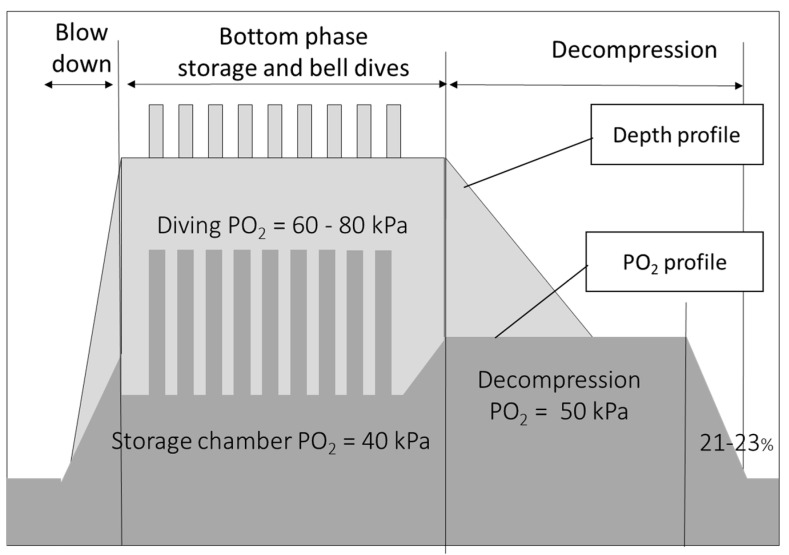
Description of the saturation in the UK sector: depth profile (compression, storage depth, bell dives, decompression) and associated PO_2_ profile.

The participants were divided as follows: 37 divers in saturation in the Norwegian project (75%) and 12 divers in saturation in the UK project (25%). The saturation duration depended on the sector regulations. It is 14 days maximum bottom time in Norway and 28 days total saturation time in the UK. The mean saturation duration was 19.70 ± 6.5 days (minimum 10 days, maximum 28 days) (see Figure 2).

### 2.4. Organizational Constraints

The voluntary divers were first involved in the study in the few hours after arriving onboard, after their pre-saturation medical examination, just before entering the saturation chambers. Baseline (control) measurements (FMD and Questionnaires) were recorded. The group of divers were then monitored during the next 12 h following the end of the decompression to surface.

The questionnaires and measurements were run in the vessel hospital room that warranted confidentiality.

**Figure 3:**
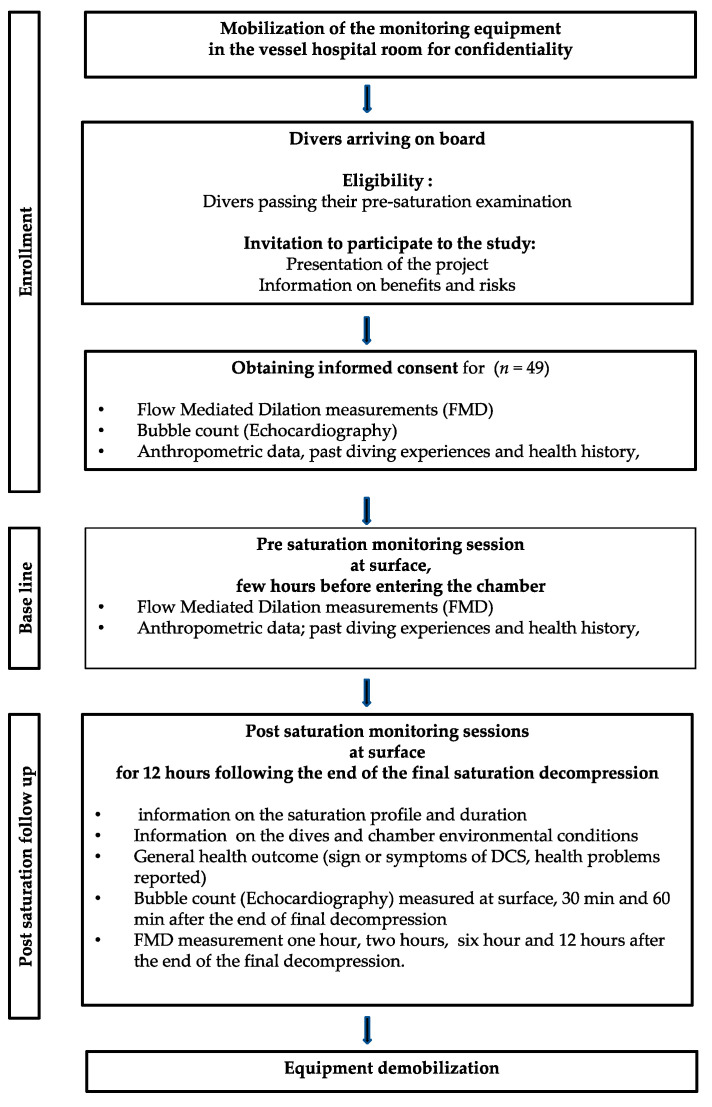
Experimental flowchart.

It is admitted that after the decompression, due to operational constraints, it was difficult to “catch” the divers at regular times and some subjects (30%) only performed one or two sessions of the four initially planned (see Figure 3).

### 2.5. Data Acquisition

#### 2.5.1. Flow-Mediated Dilation (FMD)

FMD, an established measure of the endothelium-dependent vasodilation mediated by nitric oxide (NO) [15], was used to assess the effect of diving on main conduit arteries. Subjects were at rest for 15-min in a supine position before the measurements were taken. Brachial artery diameter was measured by means of a 5.0–10.0 MHz linear transducer M-Turbo portable echocardiograph (Sonosite M-Turbo, FUJIFILM Sonosite Inc., Amsterdam, The Netherlands) immediately before and 1-min after a 5-min ischemia (induced by inflating a sphygmomanometer cuff placed on the forearm to 180 mmHg as previously described [16].

All ultrasound assessments were performed by an experienced operator, with more than 100 scans/year, which is recommended to maintain competency with the FMD method [17].

When the images were chosen for analysis, the boundaries for diameter measurement were identified manually with an electronic caliper (provided by the ultrasonography proprietary software) in a threefold repetition pattern to calculate the mean value. In our laboratory, the mean intra observer variability for FMD measurement for the operator recorded the same day, on the same site and on the same subject was 1.2 ± 0.2%.

FMD were calculated as the percent increase in arterial diameter from the resting state to maximal dilation.

#### 2.5.2. Post Saturation Diving Decompression Vascular Gas Emboli (VGE)

The echocardiographic VGE signals over the 1 min recording were evaluated by frame-based bubble counting as described by Germonpré et al. [18], but also scored according to the Eftedal-Brubakk categorical score [19].

Echocardiography was performed with a M-Turbo portable echocardiograph (Sonosite M-Turbo, FUJIFILM Sonosite Inc, Amsterdam, The Netherlands) used in a medical clinic included in the vessel while the patient was comfortably lying in a medical bed (Left Lateral Decubitus); four chamber view echocardiography loops were recorded on hard disk for offline analysis by three blinded evaluators. VGE numbers were counted at 30 min and 60 min post saturation decompression.

Evaluation of decompression stress and of the potential benefit of preventive measures has been done historically based on the presence or absence of clinical symptoms of DCS. However, for obvious ethical reasons, this is not acceptable in the field of recreational or professional diving [20]. Although imperfect, it is now accepted that research projects can use VGE data as a surrogate endpoint [6,21]. Different methods of detection of VGE are possible, such as Doppler ultrasonic bubble detectors or 2D cardiac echography [22]. During field studies, bubbles are usually detected in the right atrium, ventricle (right heart), and pulmonary artery. Then, the amount of detected VGE is graded according to different systems, either, categorical [19], semi-quantitatively [18] or continuous [21,22].

## 3. Statistical Analysis

The normality of data was performed by means of Shapiro–Wilk or D’Agostino-Pearson tests.

When a Gaussian distribution was assumed, and when comparisons were limited to two samples, paired or non-paired t-test were applied. If the Gaussian distribution was not assumed, the analysis was performed by means of a non-parametric Mann-Whitney U test or, a Wilcoxon paired test. Taking the baseline measures as 100%, percentage changes were calculated for each diver, allowing for an appreciation of the magnitude of change rather than the absolute values (one sample t-test). All statistical tests were performed using a standard computer statistical package, GraphPad Prism version 5.00 for Windows (GraphPad Software, San Diego, CA, USA).

A threshold of *p* < 0.05 was considered statistically significant. All data are presented as mean ± standard deviation (SD).

Sample size was calculated setting the power of the study at 95%, and assuming that variables associated with diving would have been affected to a similar extent as that observed in our previous studies [16,17,18] our sample reached 99%.

The linear regression line was performed using the least squares method and the lateral bands represented are in the 95% predictivity range.

## 4. Results

### 4.1. Vascular Gas Emboli

A very low number of bubbles were found in the participants after their decompression during their “bend-watch” period (the first 9 h).

Among all divers (*n* = 49), only three showed circulating gas emboli according to the EB scale that represented 0.2 ± 0.05 (mean ± SD) bubbles per heartbeat, which represents less than grade 1 on the EB grading scale in three divers. This is extremely low and doesn’t allow statistical analysis. To allow the reader to compare with other diving situations this grading is 10 times lower than an average number of bubbles after a simple dive of 25 min at 25 m considered within safety limits [23].

### 4.2. Flow Mediated Dilation

**Figure 4:**
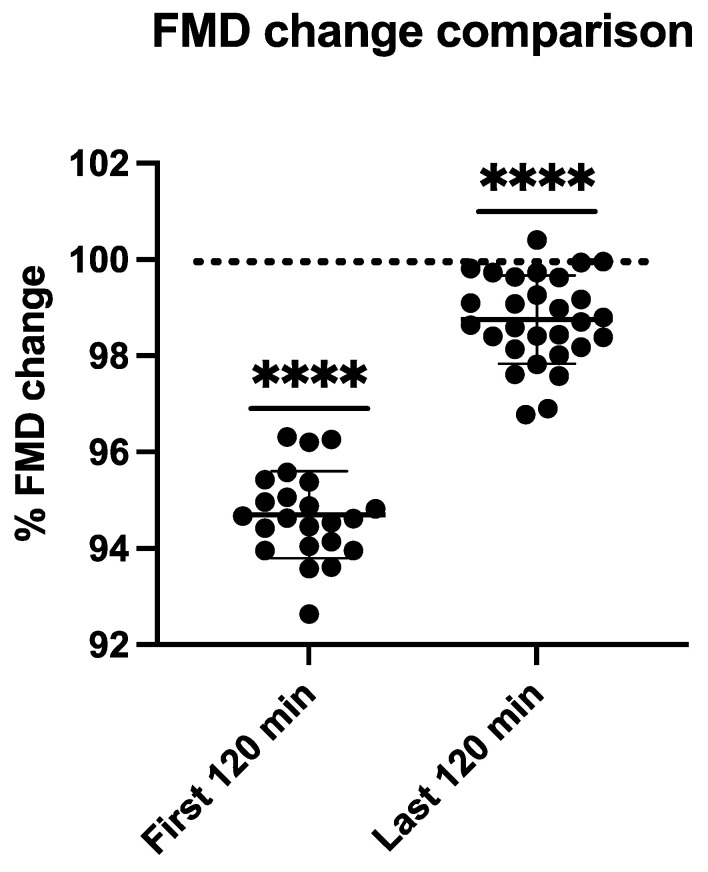
Bar graph illustrating FMD changes during the first 2 h (First 120 min.) (*n* = 23) and last 2 h (*n* = 29) (7–9 h) after saturation decompression (**** = *p* < 0.0001) (One sample t-test). (FMD Changes are presented compared to predive values represented by the dotted line at 100%).

FMD comparison between pre/post dive situation and control values is shown in Figure 4. Flow Mediated Dilation is calculated as the percentage increase of arterial diameter after an occlusion period (5 min); this post occlusion dilation was normal in our divers in pre-dive situations (107.15 ± 6.6%). After vascular occlusion, the dilation provoked by the imposed shear stress was around 7–10%. Taking the individual FMD of each diver as the baseline, the percentual mean reduction reaches 94.7 ± 0.9 % (*p* < 0.0001) during the first two hours after decompression and quickly recovers reaching 98.75 ± 0.91 (*p* < 0.0001) in the last two hours (6–8 h after decompression) (see Figure 4).

**Figure 5:**
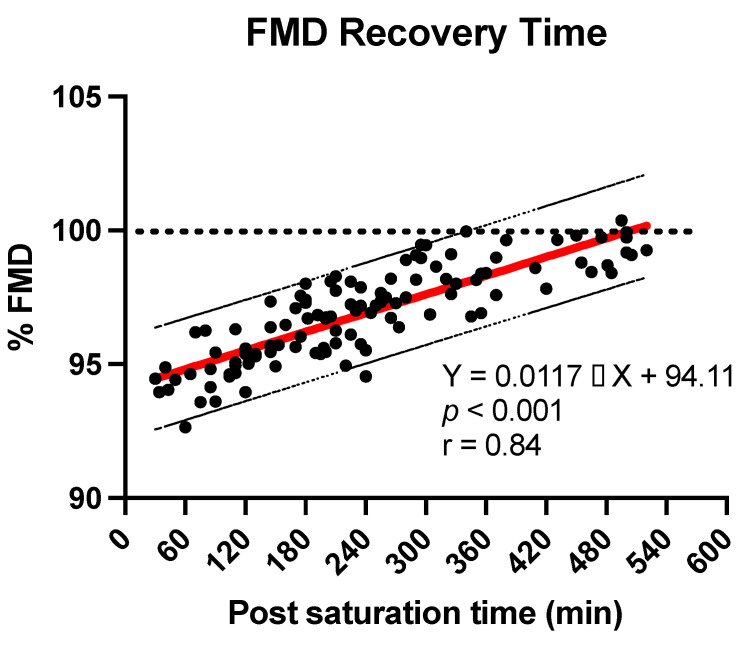
FMD evolution after exiting saturation the linear solution has been selected as the best fit approach, and the dotted lateral bands represent the 95% prediction bands.

Our data suggest that total vascular function recovery has not yet reached 8 h after the end of decompression. We then computed a best fit equation to extrapolate the time needed to achieve recovery. The linear regression line and the equation are shown in Figure 5.

## 5. Discussion

Few scientific studies have been performed in real commercial saturation conditions during the last ten years. These studies are difficult because of the offshore constraints and project planning that do not allow much time for scientific testin—not to mention the cost of accommodating the scientific team onboard. Available studies are related to the subjective evaluation of saturation operations by the divers themselves [1]. More advanced studies such as evolution of plasma or blood derived measurements have been conducted [2,24]. Given the difficulties to achieve blood sampling, other studies are conducted based on salivary, urine, epithelial, or other minimally invasive sampling techniques [24,25,26,27].

Our goal in this experiment was to document vascular recovery post saturation diving (after decompression). Vascular gas emboli are probably involved in the post dive reduction of FMD. Nevertheless, the available literature refrains us to draw a direct link between FMD reduction and VGE, since micro and macro vascularization react differently [28], and different preconditioning procedures before diving have specific actions independently on FMD and VGE, while others interfere with both [29].

In a recent experiment, a similar reduction in FMD was found in a setting excluding bubble formation, but a significant change in FMD was demonstrated depending on the oxygen partial pressure of the breathed gas [30].

Moreover, in this experimental setting, we only saw minimal levels of bubbles allowing for neglecting this stressor in such saturation decompression procedures. Decompression bubbles are very likely not to be found post decompression after saturation diving. Further investigations are needed to monitor bubbles production after excursions while being in saturation or during the decompression phase.

A nitric oxide (NO) mediated change in the surface properties of the vascular endothelium favoring the elimination of gas micronuclei has previously been suggested to explain this protection against bubble formation [31]. It was shown that NO synthase activity increases following 45 min of exercise, and, if done before a dive, it reduces VGE [32]. In saturation, although work can be considered as an exercise, it should be considered that the divers are otherwise sedentary.

It appears that FMD seems more linked to oxygen partial pressure changes during diving, whereas VGE are more depending on preexisting gas micronuclei population [33,34] in the tissues and vascular system and coping with inflammatory responses [29,35].

FMD is a marker of endothelial function and is reduced in the brachial artery of healthy divers after single or repetitive dives [29,35]. This effect does not seem to be related to the amount of VGE and was partially reversed by acute and long-term pre-dive supplementation of antioxidants, implicating oxidative stress as an important contributor to post-dive endothelial dysfunction [36,37].

Decreased nitroglycerin-mediated dilation after diving highlights dysfunction in vascular smooth-muscle cells as possible etiology of those results [37].

Very recent data show that the FMD reduction encountered after a single dive without the presence of VGE, is comparable to the reduction found with the presence of VGE [30].

The divers that volunteered in our saturation experiment were taking some antioxidant “medication” (see Table 3) as a protective measure, the trend of our data doesn’t show a clear inflexion for some participants that could be explained by antioxidants intake, although 60% of the divers declared doing so.

A recent manuscript [25] shows very interesting results allowing for following the oxidative defenses status post saturation. Although the depth and duration differ from our setting, the recovery time for NOx is around 24 h.

Our data are in tune with the NOx returning to baseline, since FMD is closely related to the availability of nitric oxide (NO), and we can see from our results that FMD almost fully recovers after 8 h. If we apply the formula extracted from our data the mean time needed to reach 100% recovery would be around 540 min (9 h) and in the least predictive range (−95%) around 600 min (10 h) would be needed to fully recover, which is confirmed by Mrakic-Sposta et al. (2020) results. In fact, their results show that 24 h post saturation, the ROS (Reactive Oxygen Species) are still significantly higher than baseline, but concomitantly TAC (Total Antioxidant Capacity) is also still high. From our results we can consider that the vascular dysfunction has already recovered and that the balance between antioxydants and prooxydants is clearly efficient and therefore fostering recovery. Another parameter measured by Mrakic-Sposta et al. [25] was IL-6 (Interleukin-6), this citokine reflects pro/anti-inflammatory response, and was increased during saturation but it was not significantly different than baseline 24 h post saturation.

## 6. Limitations

Strengths:

- This study builds on established modern methods of evaluation of decompression stress including vascular function and current theories of VGE generation.
- As there is possible large inter-individual variation for VGE and FMD effects after diving, the subjects served as their own controls.
- The measured effects are consistent with the theoretical rationale and do not require complicated new hypotheses.
- The equipment used for these experiments is readily available and reliable, inviting other research groups to repeat the study.
- The study was performed in real operational activities.
- A large number of divers volunteered for the study (never a saturation diving study addressed so many participants).

Weaknesses:

- The subjects were not homogenous or necessarily similar in body composition (age, weight, fat/lean mass distribution, sex).
- Operational constraints sometimes altered the planning of the measurements.
- Gender balance was impossible to reach.

## 7. Conclusions

This monitoring session has no equivalent in the commercial diving industry because of its duration (6 month), conditions (a working diving support vessel) and the large number of divers who volunteered for the study. It was the first time that the possibility for assessing onsite the vascular function of divers was offered during actual saturation diving operations. The study not only confirmed the role of inflammation and oxidative stress in saturation diving but it also permitted to obtain an estimation of the recovery time needed.

The lessons learnt from this experiment were that (1) scientific studies are possible even on a diving support vessel during operations under extreme environmental conditions. (2) Both national safety rules seem to provide health of the divers. (3) The equipment selected for the study was too heavy to be easily mobilized, and it could only work at ambient pressure and required a specific expertise. The future monitoring sessions, if any, should aim at using simpler equipment, which could be operated by the divers themselves inside the chamber, under pressure. Future experiments should include pressure resistance bubble measuring devices such as the O’Dive system tool to ascertain a minimal bubble number in the sub-clavian vein during excursion dives and during decompression [38].

## Figures and Tables

**Table 1:** Participants anthropometric parameters (*n* = 49).

	Mean ± SD
Age	45.7 ± 7.32
Height	180.4 ± 7.2 cm
Weight	86.4 ± 11.5 kg
BMI	26.5 ± 2.4

**Table 2:** Participants diving experience (commercial experience includes Saturation experience).

	Mean ± SD
Experience as a commercial air diver	21.3 ± 8.3 years
Experience as a saturation diver	14.7 ± 8.1 years

**Table 3:** Group antioxidant supplement intake (free administration).

Antioxidant Supplements	Yes	No	Sometimes
During normal surface life	58%	38%	4%
During saturation	59%	29%	12%

**Table 4:** Participants’ usual physical activities.

Type of Physical Activity	Percentage
Outdoor, intense like running, surfing, cycling, climbing, biking, kitesurf	72.9%
Outdoor, moderate like golf, hiking	6.8%
Indoor, intense: swimming, hockey, boxing, gym	13.6%
Moderate, or no sport	5%
Unclassified (i.e., working as a farmer)	1.7%

## Data Availability

Data are available at request from the authors.

## Abbreviations

NO	Nitric Oxide
TAC	Total Antioxidant Capacity
FMD	Flow-Mediated Dilation
HR	Heart Rate
ROS	Reactive Oxygen Species
NOx	Nitric Oxide Metabolites
NO	Nitric Oxide
DSV	Diving Support Vessel
EB	Eftedal – Brubakk Score
hPa	Hecto-Pascal
msw	Meters of sea water
VGE	Vascular Gas Emboli
IL-6	Interleukin-6
